# Monoclonal antibodies for the treatment of squamous cell carcinoma: A literature review

**DOI:** 10.1002/cnr2.1802

**Published:** 2023-04-12

**Authors:** Amirhossein Tamimi, Atena Tamimi, Fatemeh Sorkheh, Saba Mardekatani Asl, Arezoo Ghafari, Arian Ghannadi Karimi, Gisou Erabi, Hossein Pourmontaseri, Niloofar Deravi

**Affiliations:** ^1^ Student Research Committee, School of Medicine Guilan University of Medical Sciences Rasht Iran; ^2^ Student Research Committee, School of Medicine Shahid Beheshti University of Medical Sciences Tehran Iran; ^3^ Student Research Committee Babol University of Medical Sciences Babol Iran; ^4^ Student Research Committee Qazvin University of Medical Science Qazvin Iran; ^5^ Student Research Committee Urmia University of Medical Sciences Urmia Iran; ^6^ Student Research Committee Fasa University of Medical Sciences Fasa Iran; ^7^ Bitab knowledge Enterprise Fasa University of Medical Sciences Fasa Iran

**Keywords:** anti‐cancer, immunotherapy, monoclonal antibody, squamous cell carcinoma

## Abstract

**Background:**

Squamous cell carcinoma (SCC) is a relatively common and heterogenous malignancy of different organs, such as the skin, esophagus, and lungs. Although most cases experience good survival with surgical methods, management of advanced types of the disease remains challenging. Several modalities, including different chemotherapy regimens and immunotherapies, have been investigated in this matter, among which Monoclonal antibodies (Mabs) are one of the most promising ones. Since the development of Mabs, they have been widely used to treat different diseases. Mabs have shown significant efficacy with high specificity along with acceptable safety, which makes them a favorable option in cancer therapy. In this article, we aimed to review the different aspects of using Mabs in SCC therapy.

**Recent Findings:**

We found that treating with different Mabs has shown excellent efficacy accompanied by acceptable safety in treating SCC of different organs. Therefore, Mabs are considered great options in the treatment of SCC, especially in advanced cases. Overall, two highly potent types of Mabs in SCC therapy are anti‐EGFR Mabs and checkpoint inhibitors, especially Cetuximab, Nimotuzumab, and PD‐1 inhibitors. Bevacizumab is also a promising option as adjuvant therapy to other modalities.

**Conclusion:**

Although some Mabs have shown promising outcomes in SCC therapy, their application as a part of cancer treatment depends on further investigations regarding cost‐effectiveness and predictors of response. FDA has approved several Mabs in SCC therapies, and Mabs may have a crucial role in this era in the near future, especially in treating head and neck and esophageal SCC and metastatic lung cancer.

## 1 INTRODUCTION

Squamous cell carcinoma (SCC) is the second most common non‐melanoma skin cancer and a common malignancy in many countries.^1^, ^2^ In the United States, around 3.5 million none‐melanoma skin carcinomas are diagnosed yearly, 700,000 of which are new cases of SCC, resulting in 2500 deaths per year.^3^ The most common risk factor for SCC is lifetime ultraviolet (UV)‐induced nuclear damage.^4^ SCC involving the head and neck area may be particularly aggressive. Oral SCC accounts for 90% of all oral malignancies.^5^

For several years, the most common treatments for SCC included surgery, chemotherapy, radiation, and interferon‐based therapies.^6^, ^7^ Rejection of common therapies in some cases of cutaneous squamous cell carcinoma (CSCC) with local contention or distant metastasis caused great doubt in traditional methods.^8^ On the other hand, many studies confirmed several serious adverse effects of surgery and chemotherapy in treating skin cancers.^7^ Therefore, discovering new weaknesses and side effects of traditional treatments has led researchers to achieve more efficient and safer anti‐cancer therapies.^9^, ^10^ Understanding the network of receptors and ligands in different cancers and their mechanism has provided promising agents to target.^11^ Studies on head and neck squamous cell carcinoma (HNSCC) demonstrated that overexpression of epidermal growth factor receptor (EGFR) increases radio‐resistance.^12^ On the other hand, proliferation and metastasis of SCC are relatively associated with specific inflammatory cytokines such as vascular endothelial growth factor (VEGF), transforming growth factor beta (TGF‐β), hepatocyte growth factor (HGF), and IL‐6.^13^, ^14^ Targeting these factors to restrict SCC has become popular in recent research.

**FIGURE 1:**
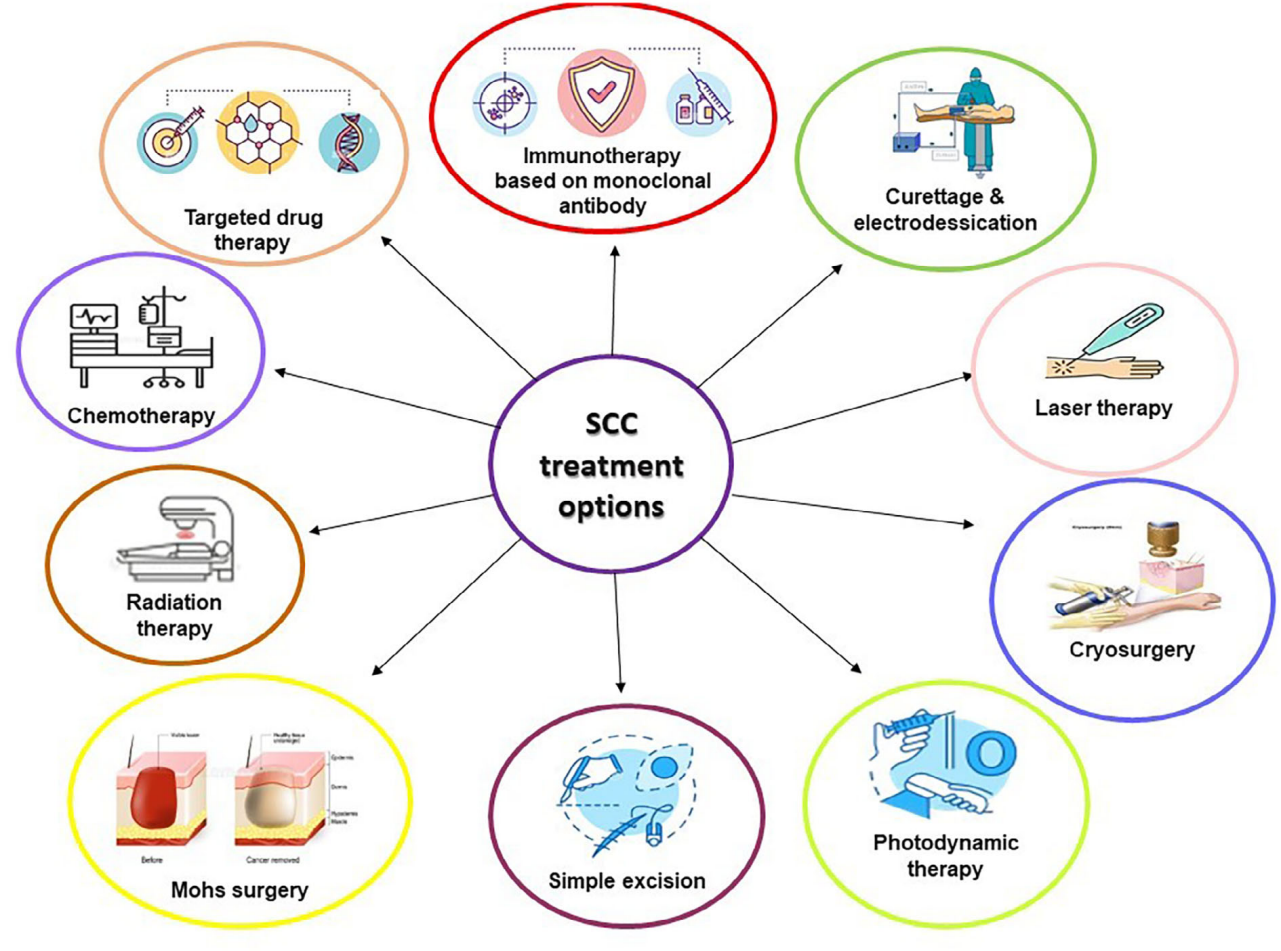
A summary of the current treatment options for SCC: main therapy for SCC includes curettage and electrodesiccation, laser therapy, cryosurgery, photodynamic therapy, simple excision, Mohs surgery, radiation therapy, chemotherapy, targeted drug therapy, and immunotherapy based on monoclonal antibodies.

The main treatment options for SCC encompass curettage and electrodesiccation, laser therapy, freezing, photodynamic therapy, simple excision, Mohs surgery, radiation therapy, chemotherapy, targeted drug therapy, and immunotherapy based on monoclonal antibodies (Figure 1).

Immunotherapy based on Mabs is considered a reliable treatment for several disorders and has become a novel component of main anti‐cancer therapies in recent years.^15^ Further studies on Mabs, such as cemiplimab, cetuximab, bevacizumab, and atezolizumab, developed different therapies to target specific ligands or receptors and prevent tumor activities.^16^ These successful findings have continued to date, and many trials have demonstrated promising anti‐cancer effects of these Mabs, such as bevacizumab and durvalumab together with or without traditional treatments.^17^, ^18^, ^19^ Here, we review the anti‐cancer properties and potential side effects of Mabs and compare their efficiency and safety.

## 2 METHOD

**FIGURE 2:**
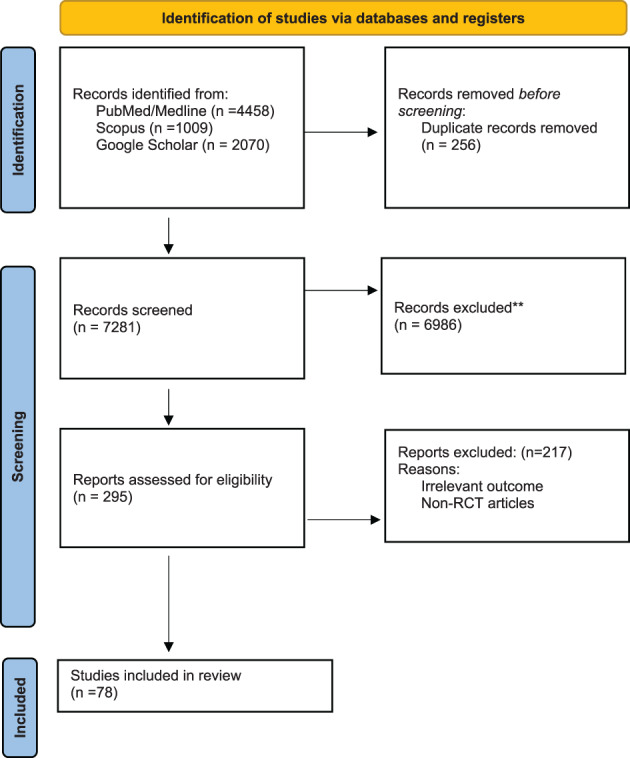
PRISMA 2022 flow diagram

We searched electronic databases of PubMed, Scopus, Web of Science, and Google scholar. We extracted articles related to Mab and SCC treatment up to June 2022. Medical Subject Headings (MeSH) terms were included. We obtained the full texts of relevant articles, and the relevant articles in the reference lists were also searched manually (Figure 2). The duplicates were removed using End note X9. Articles with irrelevant titles and studies other than clinical trials were removed. In the following, we will describe the mechanisms of Mabs in treating SCC (Table 1).

**TABLE 1:** Search strategy in databases.

Database	Search phrase	Date
SCOPUS 1009 (Medicine)	(TITLE (monalizumab) OR TITLE (ipilimumab) OR TITLE (cemiplimab) OR TITLE (durvalumab) OR TITLE (vorinostat) OR TITLE ( nivolumab) OR TITLE ((ficlatuzumab) OR TITLE ( mehd7945a) OR TITLE (onartuzumab) OR TITLE (bevacizumab) OR TITLE (nimotuzumab) OR TITLE (panitumumab) OR TITLE (zalutumumab) OR TITLE (cetuximab) AND TITLE (SCC) OR TITLE (antibody) OR TITLE (immunotherapy) OR TITLE (anti‐cancer) OR TITLE (squamous AND cell AND carcinoma) OR TITLE (monoclonal‐antibody))	6/4/22
PUBMED 4,458 results	(((((((((((((((((monalizumab[Title]) OR (Ipilimumab[Title])) OR (Cemiplimab[Title])) OR (Durvalumab[Title])) OR (vorinostat[Title])) OR (nivolumab[Title])) OR (Ficlatuzumab[Title])) OR (MEHD7945A[Title])) OR (onartuzumab[Title])) OR (Bevacizumab[Title])) OR (Nimotuzumab[Title])) OR (panitumumab[Title])) OR (zalutumumab[Title])) OR (cetuximab[Title])) AND (Squamous cell carcinoma[Title])) OR (Monoclonal antibody[Title])) OR (Immunotherapy[Title])) OR (Anti‐cancer[Title])	6/4/22

## 3 MONOCLONAL ANTIBODIES

The immune system's ability to recognize foreign cells enables the body to defend against cancerous cells.^20^ Hybridoma technology, the invention of Milstein and Kohler, brought the availability of Mabs.^21^ Immune‐based therapies, Mabs, predominantly aim to strengthen immune responses against tumor cells. Different strategies have been suggested in the last decades to stimulate T cells in favor of targeting tumor cells, such as enhancing innate immune activity and inhibiting suppressive pathways.^22^, ^23^ The mechanism by which Mabs treat cancer cases is by presenting tumor cells to destructive processes of the immune system.^16^ Mabs can activate complement‐dependent cytotoxicity (CDC) via binding to complement C1q to start the formation of the complement cascade, which can eventually destroy aimed cells. Mabs can also induce antibody‐dependent cellular cytotoxicity (ADCC), which results in the eradication of the target cell as well.^24^ In general, agglutination, inactivation of signaling proteins, or occupation of receptors are the fundamental mechanisms the antibodies employ to protect the body against cancer.^25^

Enhancing knowledge about the mentioned mechanisms has provided several potential occasions to target tumor pathogenesis. The growth factor receptors activate along with the tumor progression. Mabs may decrease tumor activities by inactivating the binding of the ligands to these receptors or by blocking signaling. This mechanism can ultimately lead to cell death induction or increased sensitivity of tumor cells to therapeutic drugs.^26^ In another condition, bevacizumab blocks vascular endothelial growth factors (VEGFs) receptors and inhibits tumor‐mediated angiogenesis.^27^ Furthermore, numerous studies suggest that Mabs play a potential role in triggering the acquired immunity against malignancies.^28^

Consequently, Mabs provided unbelievable oncological purposes in several approaches.^29^ In addition, many studies approved their therapeutic applications in other disorders, such as inflammation, infection, and cardiovascular disease.^30^ Therefore, many recent trials introduced Mabs as an effective therapeutic approach in cancer treatments.^31^

Using Mabs alone or in combination with other methods showed encouraging outcomes in controlling SCC.^32^ Cetuximab, for instance, has been used in both directions to treat head and neck squamous cell carcinoma (HNSCC) and proven its efficacy.^33^, ^34^ The average half‐lives of Mabs last for 18–21 days. A reduction in half‐lives might occur due to adverse binding to unspecific targets.^35^ Ectodomain shedding of Mabs receptors is another issue which seems to hinder reaching the target cells.^36^ Antidrug antibodies (ADAs) generation can also be a result of provoking the patient's immune system.^37^ However, chemotherapy seems to leave harsher side effects.^38^ Altogether, Mabs are known to function through various mechanisms in treating SCC. Some Mabs target EGFR, HER2, IGF‐1R, HGF, and VEGF; others may be checkpoint inhibitors (including Mabs to PD‐1, CTLA4, and NKG2A) or tumor‐targeting Mabs such as U36 and Mab E48. In the following pages, we reviewed the Mabs in SCC immunotherapy. Table 2 summarizes the data of the included studies.

**TABLE 2:** Summary of clinical trials on monoclonal antibodies

Type of mAb	Mechanism of inhibition	Scc type	Outcomes	Reference
Avelumab	Anti‐programmed death ligand 1 antibody	R/M SCCHN	Median PFS: 1.4 (1.8) months OS: 8.0 months ORR: 9.2% (13.1%) CBR:	149
Avelumab, Cetuximab	PD‐L1 and EGFR inhibitors	Advanced or metastatic squamous cell anal carcinoma	ORR = 10% (95% CI, 2.1%–26.5%) vs. 17% (95% CI, 5.6%–34.7%), respectively. Disease control rate = 50% (95% CI, 31.3%–68.7%) vs. 57% (95% CI, 37%–74.5%). At a median follow‐up of 26.7 months (IQR 26.5%–26.9%), median PFS = months (95% CI, 1.8–4 months) vs. 3.9 months (95% CI, 2.1–5.6). median OS = 13.9 months (95% CI, 7.7–19.4) vs. 7.8 (95% CI, 6.2–11.2)	52
Bevacizumab	Anti‐VEGF	Recurrent or persistent squamous cell carcinoma of the cervix	Median OS: 7.29 months Median PFS: 3.40 months Median RD: 6.21 months	87
		(R/MHNSCC)	Median OS: 11.3 months Median TTP: 5 months ORR: 30% CRR: 5% Disease control: 86%	94
		Locally AHNSCC	3‐year OS: 82% 3‐year PFS: 71% Overall RR: 95%	91
		Locoregionally advanced nasopharyngeal carcinoma	2‐year LPFI: 83.7% 2‐year OS: 90.9% 2‐year PFS: 74.7% 2‐year DMFI: 90.8% 2‐year LPFI: 83.7%	195
		Locally and/or regionally (AHNSCC)	2‐year OS: 88% 2‐year PFS: 75.9%	93
		Locoregionally AHNSCC	Mean survival: 61.3 months DCE‐CT contrast enhancement, tumor proliferation, and tumor hypoxia decreased remarkably after the bevacizumab solo therapy period. Tumor hypoxia and proliferation had a bigger reduction during the combination therapy.	95
		VSCC	CR: 22.2% Median duration of treatment: 7 months SD: 33%	96
Cemiplimab	PD‐1 checkpoint inhibition	Advanced cutaneous squamous‐cell carcinoma	Phase 1: Independent central review assessed RR = 50% Durable DCR = 65% Phase 2: Estimated probability of PFS at 12 months: 53% Estimated probability of OS at 12 months: 81%	153
		Unresectable locally advanced or metastatic cutaneous squamous cell carcinoma	Independent central review assessed ORR: 50.0% Durable DCR: 65.4%	196
		Stage III/IV (M0) cutaneous squamous cell carcinoma of the head and neck (CSCC‐HN)	ORR: 30% pCR: 55% MPR: additional 15% patients No case of relapse had been found with a 3.8‐month median follow‐up	152
		locally AHNSCC	Complete response was seen in 13% of patients, and 31% of patients experienced a partial response	197
		Metastatic cutaneous squamous cell carcinoma	ORR per ICR: 41.1% (G3) vs. 49.2% (G1) and 45.2% for both groups combined. Estimated progression‐free probability at 12 months: 52.5% Estimated survival probability at 12 months: 80.6%	198
		CSCC mCSCC	The median duration of follow‐up: 15.7 months. ORR per ICR: 46.1% CR: 20.3%, 12.8%, and 16.1% for groups 1, 2, and 3 OR: 87.8% OS: 73.3%	154
		R/M SCCHN	PR: 6.7% DCR: 40.0% Median progression‐free survival: 1.8 months	155
		Advanced cutaneous squamous cell carcinoma (CSCC)	Median follow‐up: 8 months ORR: 67% CR: 33% PR: 33% DCR: 72%	156
		Advanced cutaneous squamous cell carcinoma (CSCC)	ORR: 45.5% RD: 5.5 months in C group/3 months in C/RT group Follow‐up period: 6 months and 1 year	157
		Advanced cutaneous squamous cell carcinoma (CSCC): locally & metastatic	PFS: 5.9 months Median follow‐up period: 9 months AE: 46% and most mild	158
		Cutaneous squamous cell carcinoma (CSCC)	Complete pathological response: 51% Major pathological response: 13% AE: 87% (regardless of the relationship to treatment) AE (grade ≥3): 18%	159
		Orbital SCC	Median duration of treatment: 5 months Median follow‐up: 15 months ORR: 69.2% Complete response: 53.8% Partial response: 15.4% SD: 7.7% PD: 15.4%	199
		LA SCC	OR: 76.7% CR: 30% AE: Fatigue: 23.3% Skin toxicity: 33.3%	200
Cetuximab	Inhibition of EGFR	Locoregionally AHNSCC	HR for disease progression or death = 0.70 HR for locoregional progression or death = 0.68 The median duration of locoregional control = 14.9 months for G1 and 24.4 months for G2 Median OS = 29.3 months for G1 and 49.0 months for G2	45
		Unresectable HNSCC	DCR at 6 weeks = 69% Best ORR = 28% mean OS = 8.1 months	175
		Inoperable cutaneous SCC	DCR = 78% (50% for CM, 87.5% for CC and 100% for CR) RR = 47% (33% for CM, 37.5% for CC and 80% for CR)	48
		R/MHNSCC	Median PFS: 4.5 months G1 vs. 2.0 months G2 (HR 0.37) Regarding QoL, OS, and toxicity there was no significant difference between groups	49
		R/MHNSCC	MOS: Locoregional recurrent: 15.6 months Metastatic: 9.7 months *p* = .004 HR: 0.64 DCR: 62.5% PFS: Locoregional recurrent: 5.8 months Metastatic: 4.2 months *p* = 0.008 HR = 0.67 DCR: 45%	201
		R/MHNSCC	MPFS: 6.5 months AE: rash and fatigue	202
		Advanced HNSCC	1‐year PFS: 51.5% 1‐year locoregional PFS: 56.1% Treatment completion rate: 77.2% AE Pharyngeal mucositis: 48.5% Radiation dermatitis: 45.6% Oral mucositis: 40.4% Pneumonitis: 7%	203
		HNCSCC	Median follow‐up period: 42 months MOS: not reached Median DFS: 64 months 2‐year OS: 75% 2‐year DFS: 70.8% AE Radiation related dermatitis: 12.5% Cetuximab related rash: 16.7%	204
		R/M Oral SCC	Best OS: 48.4% DCR: 61.3% MPFS: 6 months MOS: 13 months AE (grade 3–4) Leukopenia: 16.1% Acne‐like rash: 12.9% Neutropenia: 9.7%	205
Cixutumumab	Inhibition of IGF‐1R	R/M HNSCC	Median PFS: 1.9 vs. 2 months Clinical benefit rate: 5.9 vs. 15.3%	78
Duligotuzumab (MEHD7945A)	Inhibition of EGFR and HER3	R/MHNSCC	Progression‐free survival: 4.2 vs. 4 months OS: 7.2 vs. 8.7 months Objective response rate: 12 vs. 14.5%	99
Durvalumab	Anti‐PD‐L1	R/MHNSCC	Median OS: 7.1 months Median PFS: 2.1 months 12‐month OS: 33.6% 12‐month PFS: 14.6% Objective RR: 16.2%; 29.4%, and 10.9% in patients with and without HPV, respectively	142
		R/MHNSCC	Median OS: 7.6 months in G1, 6.0 months in G2, and 5.5 months in G3	144
		R/MHNSCC	Median OS: 8.4 months Median PFS: 1.4 months Objective RR: 6.5%	143
		R/MHNSCC	Median OS: 6.5 months in G1, 7.6 months in G2, and 8.3 months in G3 Overall RR in G1: 18.2% in G1, 17.9% in G2, and 17.3% in G3 12 months OS: 30.4% in G1, 37.0% in G2, and 30.5% in G3	206
		R/MHNSCC	G1 was similar to G2 and G3 in OS. 12‐month survival was 30.4% in G1, 37.0% in G2, and 30.5% in G3	18
		Locally AHNSCC	1‐year OS: 60% 1‐year PFS: 63.8%	207
		HNSCC	In the HAWK study (111 patients), the median OS was 7.1 months (95% confidence interval [CI], 4.9–9.9), and in the CONDOR study, it was 6.0 months (95% CI, 4.0–11.3 months) among patients who received durvalumab monotherapy (65 patients). In the EAGLE study, 237 durvalumab‐treated patients had a median OS of 7.6 months (95% CI, 6.1–9.8 months). Among the 111 HAWK patients, 106 had evaluable biomarker data, with CONDOR (52 patients out of 65) and EAGLE (209 patients out of 237) having slightly larger proportions of missing biomarker data. A log‐rank test revealed that the OS for durvalumab‐treated patients with evaluable biomarker data was similar in all three studies (*p* = .74)	146
		Esophageal squamous cell carcinoma	86 participants were randomly assigned to the durvalumab (*n* = 45) or placebo (*n* = 41) arm between March 2016 and June 2018. The average period of follow‐up was 38.7 months. There was no difference in DFS [hazard ratio (HR) 1.18, 95% confidence interval (CI) 0.62–2.27, *p* = 0.61] or overall survival (HR 1.08, 95% CI 0.52–2.24, *p* = 0.85) between the two arms. Patients whose post‐CCRT programmed death‐ligand 1 (PD‐L1) expression profile could be measured (*n* = 54) underwent subgroup analysis. Durvalumab was related to prolonged overall survival relative to placebo (36‐month survival rate: 94% versus 64%; HR 0.42, 95% CI 0.10–1.76), in the PD‐L1‐positive group based on tumor proportion score of ≥1%	145
Ipilimumab	Targeting CTLA‐4	Advanced squamous non‐small‐cell lung cancer	OS and PFS were not statistically different between study groups. ORR: 44% (including one CR) and 47% (including two CRs) Ipilimumab + chemotherapy showed more median duration of response	161
		Metastatic or recurrent human papillomavirus‐related cervical carcinoma	Median PFS = 2.5 months Median OS: 8.5 months Negative PD‐L1: 71%	180
Ipilimumab & Nivolumab	Targeting CTLA‐4	Untreated oral cavity squamous cell carcinoma	Volumetric response: 50% G1 and 53% G2 RECIST response: 13% G1 and 38% G2 Clinical to pathologic down staging: 69% G1 and 53% G2	163
Monalizumab	Inhibition of binding of CD94‐NKG2A to HLA‐E	R/M SCCHN	ORR: 25% Partial response: 31% Stable disease: 54% Progressive disease: 11%	169
Necitumumab	Anti‐EGFR	Lung SCC	On the 8th day of the second cycle, severe hypomagnesemia (asymptomatic grade 4) occurred. By magnesium replenishment, hypomagnesemia was improved; however, tumor progression was detected during the magnesium correction period	70
Nimotuzumab	Targeting EGFR	HNSCC	Overall response (100% vs. 70%), PFS (56.52% vs. 21.74%), OS (69.57% vs. 21.74%) increased in group I compared to control group. Overall response (76% vs. 40%), PFS (34.78% vs. 13.04%), OS (39.13% vs. 21.74%) increased in group II compared to control group	176
		HNSCC	Nimotuzumab improved median OS, compared to placebo (12.50 months vs. 9.47 months). Besides, patients in group A had higher median survival (14.0 months) than group B (8.83 months). Overall, the efficiency of Nimotuzumab was related to the level of EGFR expression	62
		AHNSCC	Adding Nimotuzumab to both CRT and RT raised ORR, PD, median 5‐year OS, and PFS significantly	61
		Advanced esophageal SCC	ORR: 51.8% DDC: 10.8 months OS: 20.2 months	208
		Esophageal SCC	Median PFS (11.0 vs. 5.8) and OS (21.5 vs. 9.7), and 1‐year survival (74.8% vs. 44.4%) were relatively higher in low expression of EGFR than in high expression	209
		Esophageal SCC	Complete RR: 11.1% Partial RR: 81.5% SD Rate: 3.7% PD Rate: 3.7% Overall ORR: 92.6% median follow‐up: 35.1 months OS: 1 year: 61.1% 2 years: 35.2% MOS: 16 months PFS: 1 year: 42.6% 2 years: 16.7% MPFS: 10 months	67
Nivolumab	Programmed death‐1 inhibitor	Oesophageal SCC	Median OS: 10.8 months Centrally assessed and investigator‐assessed median durations of PFS were 1.5 and 2.3 months. Immune‐related objective response rate and DCR: 25% and 67% median immune‐related PFS: 2.9 months	210
		R/MHNSCC	Median OS: 7.7 months G1 and 5.1 months G2 The Kaplan–Meier‐estimated 24‐month OS rate: 16.9% G1 and 6.0% G2 ORR: 13.3 G1 vs. 5.8 G2 median Time to response: 2.1 months G1 and 2.0 months G2 median Duration of response: 9.7 months G1 and 4.0 months G2	118
		R/MHNSCC	In patients with prior cetuximab exposure: the median OS: 5.1 months for G2 and 7.1 months for G1 (HR, 0.84) In patients without prior cetuximab exposure: the median OS: 4.9 months for G2 and 8.2 months for G1 (HR, 0.52) Significant improvement in OS	178
		Advanced oesophageal SCC	Median OS: 8.4 months for G2 and 10.9 months for G1 (HR 0.77)	117
		R/M oral SCC	Overall response: 27.3% DCR: 42.6% MPFS: 2.7 months 1 year: 25.4% 2 years: 19.2% OS: 11.2 months 1 year: 47.3% 2 years: 33.6% AE (grade ≥ 3): 5.5%	122
		Advanced oesophageal SCC	CPS < 1 Median PFS: 1.5 HR vs. CPS ≥ 10: 1.67 ORR: 0% DCR: 12% CPS = 1–10 Median PFS: 2.5 HR vs. CPS ≥ 10: 1.33 ORR: 25% DCR: 50% CPS ≥ 10 Median PFS: 3.2 ORR: 30% DCR: 60%	123
		R/M HNSCC	MPFS Platinum‐refractory: 2.7 months Platinum‐sensitive: 5.3 months *p* = .03 OS platinum‐refractory: 8.8 months Platinum‐sensitive: 17.1 months *p* = .06 DCR: *p* = .11 ORR: *p* > .99 AE: *p* > .99	211
		R/M HNSCC	Median follow‐up periods OS: 73.1 weeks PFS: 48.1 weeks MOS: 74.1 weeks MPFS: 18.5 weeks 2‐year survival rate: 33.4% 2‐year PFS rate: 22.5% Best overall response rate in Complete response: 10.2% Partial response: 19.3% SD: 25% PD: 44.3% AE: 40 AE in 34%	212
		Advanced/MCSCC	Best ORR: 58.3% No complete response Median follow‐up: 17.6 months MPFS: 12.7 months MOS: 20.7 months AE Any grade: 87.5% Grade ≥ 3: 25%	213
		ESCC	ORR: 17.2% OS rate: 3 years: 10.9% 5 years: 6.3% AE: 63.1%	214
		R/MHNSCC	CBR: 66.7% MPFS: 3 months MOS: 8 months AE: 8.3% (GI)	215
		MHNSCC	OPP N: 34.5% N + SBRT: 29% *p* = .86 OS: *p* = .75 PFS: *p* = .26 Response duration: *p* = .26 AE (Grade 3–5) N: 13.3% N + SBRT: 9.7% *p* = .7	216
		Recurrent resectable HNSCC	AE (grade ≥ 3): 11% Pathologic response: 43% Median follow‐up: 22.8 months DFS 1 year: 55.2% 2 years: 64% OS 1 year: 85.7% 2 years: 80%	217
Onartuzumab	Inhibition of MET receptor	AHNSCC	The ORR: 40% for the onartuzumab arm and 43.4% for the placebo arm ITT population (median PFS 4.9 months in both arms, median OS, respectively, 9.1 months vs. 8.5 months) MET IHC^+^ population (median PFS 5.0 vs. 5.2 months‐ median OS 10.8 vs. 7.9 months)	82
Panitumumab	Inhibition of EGFR	R/MHNSCC	Median OS = 9.0 months in the control group and 11.1 months in the panitumumab group (HR 0.873). Median PFS = 4.6 months in the control group and 5.8 months in the panitumumab group (HR 0.780). In patients with p16‐negative tumors, Median OS was longer in the panitumumab group than in the control group (11.7 months vs. 8.6 months) No significant difference between groups in terms of Median OS in patients with p16‐positive tumors. In the control group, p16‐positive patients had numerically, but not statistically, longer OS than p16‐negative patients (HR 0.70).	56
		Unresected, locally AHNSCC	Local‐regional control at 2 years was 51% in the radiotherapy plus panitumumab group and 61% in the chemoradiotherapy group	58
		Unresected, locally AHNSCC	Local‐regional control at 2 years was 61% in G2 and 68% in G1. No significant differences in the results	57
Pembrolizumab	Inhibition of PD‐L1	R/MHNSCC	ORR: 18% by central imaging vendor and 20% by investigator review OSR: 59% in 6 months Progression‐free survival: 23% in 6 months	129
		R/MHNSCC	ORR: 18% in all patients, 25% in HPV‐positive patients, 14% in HPV‐negative patients	130
		R/MHNSCC	ORR: 16% (95% confidence interval 11, 22) MRD: 2.41 to 27.71 months	218
		HNSCC	ORR: 18% MRD: range, 2+ to 30+ months; 85% of responses lasted ≥6 months OSR: 38% in 12 months	124
		R/MHNSCC	ORR: 19% (95% confidence interval, 7%–39%) MFD: 12 months (range, 2–21 months) MOS: 11.6 months (95% confidence interval, 4.7–17.7 months)	131
		Metastatic esophageal squamous cell carcinoma (ESCC)	MFD: 5.8 months ObRR: 9.9% among all patients, 14.3% among patients with ESCC, 5.2% among patients with adenocarcinoma, 13.8% among patients with PD‐L1–positive tumors, 6.3% among patients with PD‐L1‐negative tumors	127
		Refractory CSCC	27‐weeks PFS: 37% MRD: 27.3 months ORR: 32% CBR: 37%	132
		ESCC	Median OS: 10.0 months for G1 and 6.5 months for G2 (*p* < .0001) Median PFS: s 2.3 months for G1 and 3.1 months for G2 (*p* = .020) ORR: 17.1% for G1 and 7.1% for G2 MDR: 10.5 months for G1 and 7.7 months for G2	133
		R/M HNSCC	6‐month ORR: 45% MDR for patients who had a complete or partial treatment response: 13.1 months	51
		LA or R/M CSCC	LA cohort, ORR: 50.0% of patients with LA CSCC and 35.2% of patients with M/R CSCC CRR: 16.7% of patients with LA CSCC and 10.5% of patients with M/R CSCC PRR: 33.3% of patients with LA CSCC and 24.8% of the patients with M/R CSCC	134
		R/M HNSCC	ORR: 18% for G1 and 14% for G2 Median PFS: 1.7 months for G1 and 2.7 for months for G2	136
		HNSCC	ORR: 24% MDR: 7 months 9‐month OS: 64.7% 9‐month PFS: 19.0% A higher response rate (44%) was reported in patients who were human papillomavirus–positive.	137
		R/M HNSCC	ORR: 41.4% Median PFS: 4.1 months Median OS: 8.9 months	138
		HNSCC	Tumor mutational burden, clonality‐weighted tumor mutational burden, and 18‐gene T‐cell‐inflamed gene expression had remarkable correlations with an objective response. Neoantigen load and PD‐L1 combined positive scores were positively correlated with clinical response.	135
		LA‐HNSCC	Median follow‐up: 25 months 15‐month locoregional control rate Cetuximab‐RT: 59% Pembrolizumab‐RT: 60% *p* = .91 PFS HR: 0.85 *p* = .47 OS HR 0.83 *p* = .49 AE (at least one grade ≥ 3) Cetuximab‐RT: 92% Pembrolizumab‐RT: 74% *p* = .006	139
Pembrolizumab And Cetuximab	Inhibition of PD‐L1	HNSCC	Pembrolizumab with platinum and 5‐fluorouracil is an effective first‐line therapy for chronic or metastatic HNSCC, and pembrolizumab alone is an acceptable first‐line therapy for PD‐L1‐positive chronic or metastatic HNSCCC, based on the effectiveness and safety detected	128
		R/MHNSCC	MOS: 8.4 months (95% CI 6.4–9.4) with pembrolizumab and 6.9 months (5.9–8.0) with a standard of care	126
	PD‐1 and EGFR inhibitor	Head and neck squamous cell carcinoma	ORR = 45% (95% CI, 28%–62%)	51
Pembrolizumab and Vorinostat	Inhibition of PD‐1	R/MHNSCC and salivary gland cancer(SGC)	In the HNSCC group: MPFS: 4.5 months MOS: 12.6 months MFD: 12.6 months In the salivary gland cancer (SGC) group: MPFS: 6.9 months MOS: 14.0 months MFD: 13.1 months	219
Pembrolizumab, standard of care (SOC)(methotrexate, docetaxel, or cetuximab)	Inhibition of PD‐1	HNSCC	HRQoL compliance for pembrolizumab: 75.3% at week 15 HRQoL compliance for SOC: 74.6% at week 15	220
Zalutumumab	Human anti‐human antibodies (HAHA) and EGFR/HER2neu Assessment	R/M SCCHN	Median OS = 5.3 months Median PFS = 2.1 months Subgroup analysis by ECOG PS revealed a median OS of 6.3 months for performance status = 0–1 and 2.5 months for performance status = 2 Objective response rate = 5.7% Disease control rate = 39.8%	54
		R/MHNSCC	No significant OS difference Tumor response rate = 6.3% (3.3–11.0) in the ZG and. 1.1% (95% CI 0.0–5.7) in the CG The disease control rate = 48%^39^, ^40^, ^41^, ^42^, ^43^, ^44^, ^45^, ^46^, ^47^, ^48^, ^49^, ^50^, ^51^, ^52^, ^53^, ^54^ in the ZG and 27%^16^, ^19^, ^20^, ^21^, ^22^, ^23^, ^24^, ^25^, ^26^, ^27^, ^28^, ^29^, ^30^, ^31^, ^32^, ^33^, ^34^, ^35^, ^36^ in the CG. The HR for death between the patients in the zalutumumab group and controls, stratified by WHO performance status: 0.77	53

Abbreviations: AE, adverse events; ALT, Alanine aminotransferase; AST, aspartate aminotransferase; CR, complete response; CRR, complete response rate; DCR, disease control rate; DFS, disease free survival; DLTs, dose‐limiting toxicities; DMFI, distant metastasis‐free interval; DOR, duration of response; EE: enhanced expression; ESCC, esophageal squamous cell carcinoma; HNSCC, head and neck squamouse cell carcinoma; HR, hazard ratio; HRQoL, health‐related quality of life; ICR, per independent central review; LA, locally advanced; LPFI, locoregional progression‐free interval; MFD, median follow up duration; MOS, median overall survival; MPFS, median progression‐free survival; MPR, major pathology response (10% viable tumor); MRD, median response duration; ObRR, objective response rate; ORR, objective response rate; OS, overall survival; OSR, overall survival rate; pCR, pathologic complete response; PD, progressive disease; PFS, progression‐free survival; QoL, quality of life; R/M, recurrent/metastatic; RD, response duration; RR, response rate; SD, stable disease; SoC, cetuximab, a fluoropyrimidine, methotrexate or a taxane; TREA, treatment‐related adverse event; TTP, time‐to‐progression.

## 4 ANTIBODIES TO RECEPTORS/LIGANDS IMPORTANT FOR SQUAMOUS CELLCARCINOMA DEVELOPMENT

### 4.1 Anti‐epidermal growth factor receptor (anti‐EGFR)

Studies have proven the role of EGFR in the pathogenesis and progression of various cancers. Such as colorectal carcinoma and pancreatic carcinoma. The overexpression of EGFR and EGF‐like peptides is common in human carcinomas, and evidence has shown that these proteins promote cell transformation *in vitro* and *in vivo*. EGFR gene amplification and EGFR tyrosine kinase domain mutation were shown to happen in patients with carcinoma. Appealingly both of these EGFR genetic changes are frequently responsive to EGFR targeting agents.^39^

#### 4.1.1 Cetuximab

Cetuximab (Erbitux2), a high‐affinity recombinant Immunoglobulin G1 (IgG1), inhibits ligand binding and promotes EGFR interiorization and downregulation.^40^ Various mechanisms are considered to cause the anti‐cancer action of Cetuximab, involving the direct blockage of EGFR tyrosine kinase, restricting cell cycle, raising the quantity and activity of the cell death‐inducing molecule, such as Bcl‐2 and Bax, and increasing radiotherapy and chemotherapy anti‐cancer effects.^41^ Besides, clinical trials proved that cetuximab inhibits invasion, angiogenesis, and metastasis.^42^

The overactivation and high expression of EGFR in almost all HNSCC cases (over 90%) have been reported.^43^, ^44^ Consequently, elevated EGFR promotes oncogenesis and plays an independent poor prognostic role in HNSCC.^44^, ^45^ Furthermore, EGFR blockage makes tumor cells susceptible to radiation; on the other hand, radiotherapy promotes the expression of EGFR.^45^, ^46^, ^47^ Therefore, EGFR‐blocking would be a good choice to improve the outcome of patients in comparison to radiotherapy alone. Previous studies showed that combining cetuximab and radiotherapy restricts HNSCC more efficiently and decreases mortality and adverse effects.^45^

Controversial findings on cetuximab efficiency and toxicity have been reported.^48^, ^49^ Recently, in a novel study, Fushimi et al. demonstrated that even though the combination of cetuximab and paclitaxel increased median progression‐free survival (PFS) and overall survival (OS) in patients with recurrent or metastatic head and neck squamous cell carcinoma (R/MHNSCC), this regime led to some adverse effects, such as rash‐acneiform, anemia, and neutropenia.^50^ Therefore, more reliable studies with larger sample sizes are required to ensure the efficiency of treating SCC patients with cetuximab.

In a non‐randomized, phase II clinical trial by Sacco et al., 33 patients with R/MHNSCC were treated with Pembrolizumab and Cetuximab regimens. The patients were at least 18 years old with no previous history of immunotherapy. Given to median follow‐up duration = 7.3 months (3.9–10.9), overall response rate was 45% (95% CI, 28%–62%).the most common adverse effect was oral mucositis, and no death occurred.^51^

In another phase II randomized clinical trial, Lonardi et al. evaluated the efficacy of Avelumab alone (arm 1, *n* = 30) in comparison to a dual combination of Cetuximab with Avelumab (arm 2, *n* = 30) in patients with aSCAC (advanced metastatic squamous cell anal carcinoma). Overall response rate were 10% (95% CI, 2.1%–26.5%) versus 17% (95% CI, 5.6%–34.7%), respectively. Disease control rate were 50% (95% CI, 31.3%–68.7%) versus 57% (95% CI, 37%–74.5%). At a median follow‐up of 26.7 months (IQR 26.5%–26.9%), median PFS were 2 months (95% CI, 1.8–4 months) versus 3.9 months (95% CI, 2.1–5.6). median OS were 13.9 months (95% CI, 7.7–19.4) versus 7.8 (95% CI, 6.2–11.2). The results emphasized the promising role of dual therapy of EGFR and PD‐L1 inhibitors in the future.^52^

#### 4.1.2 Zalutumumab

Machiels et al. showed that treating SCC with zalutumumab, a fully human IgG1 Mab against EGFR, inhibits tumor progression without any signs of adverse effects like rashes.^53^ following these results, another study showed that zalutumumab's safety and efficiency in controlling R/MHNSCC are acceptable.^54^ Data from Phase I and II trials proved this novel Mab as a safe and promising treatment for SCC; also, recent Phase III studies support previous findings.^55^

#### 4.1.3 Panitumumab

Panitumumab, a recombinant human IgG2 Mab to EGFR, showed successful results in restricting R/MHNSCC without significant adverse effects.^56^ However, despite initial positive results, several studies demonstrated that panitumumab could not be a reliable therapy.^57^, ^58^, ^59^ Eventually, evaluating panitumumab safety and efficacy in recent studies, Necchi et al. showed that treating SCC with this Mab would show good curative effects on cancer.^60^ In conclusion, due to deep controversies in combining Panitumumab with other treatment options, we have a long way to approve its value in restricting HNSCC.

#### 4.1.4 Nimotuzumab

In 2013, Reddy et al. used nimotuzumab and humanized IgG1 antineoplastic Mab with CRT/RT as a viable therapeutic option in patients with HNSCC, and introduced this combination as a novel therapeutic choice.^61^ Moreover, in the last decade, further studies supported these results.^62^, ^63^, ^64^ Consequently, Si et al. showed that using Nimotuzumab along with chemotherapy is a potential method and efficient therapy for skin cancers, even as a first‐line treatment of SCC.^65^ In addition, in some cases, Nimotuzumab showed a significant improvement in the OS rate in patients with locally advanced or metastatic esophageal squamous cell carcinoma (ESCC).^66^

A study was performed by Zhang et al., in which the effect of nimtuzumab in addition to chemo/radiotherapy for the treatment of unresectable esophageal SCC was investigated. The overall objective response rate was reported to be 92.6% and the patients were followed up for 35.1 months, during which the 1 year, 2 years and median overall survival rates were 61.1%, 35.2%, and 16 months, respectively. Also, 1‐year, 2‐year progression‐free survival and median progression‐free survival were evaluated as 42.6%, 16.7%, and 10 months, respectively.^67^

#### 4.1.5 Necitumumab

In a phase 3 randomized controlled trial by Thatcher et al., necitumumab, recombinant human IgG1 Mab binding to EGFR, has shown promising results in the treatment of stage IV squamous non‐small‐cell lung cancer patients. It improved the overall survival of these patients, accompanied by acceptable safety.^68^ However, it was not consistent with another trial on this topic by Paz‐Ares et al.^69^

Nakao et al. reported severe hypomagnesemia in a 72‐year‐old man with Hashimoto's disease, old MI, disseminated nontuberculous mycobacteriosis, and lung SCC. He managed to survive for more than 3 years, but tumor progression was detected, after using Nivolumab as the fifth line of treatment. In the sixth line of treatment, cisplatin and gemcitabine combined with Necitumumab were used. On the eighth day of the second cycle, severe hypomagnesemia occurred, one of the documented adverse effects of monoclonal antibodies.^70^

As a sixth‐line treatment, a 72‐year‐old man with Hashimoto's disease and advanced lung squamous cell carcinoma was given cisplatin plus gemcitabine with necitumumab, which is a human monoclonal antibody that interacts with EGFR. Tumor decrease was detected, but on day 8 of the second cycle, asymptomatic grade 4 hypomagnesemia occurred. On day 40, he received magnesium replenishment, and his hypomagnesemia improved. However, tumor development was seen over the magnesium correction period. Although hypomagnesemia is a documented side effect of anti‐EGFR antibody therapy, there have been no case reports of severe hypomagnesemia or its clinical course. Pembrolizumab monotherapy, carboplatin plus nab‐paclitaxel, docetaxel plus ramucirumab, tegafur/gimeracil/oteracil (S‐1) therapy, and nivolumab therapy were all used to treat him.

Even though he had attained long‐term survival (>3 years), tumor growth was noticed shortly after starting nivolumab treatment. Nakao et al. opted to use cisplatin with gemcitabine in conjunction with necitumumab (every 3 weeks on days 1 and 8) therapy because his performance status was 1 at the end of nivolumab therapy as a fifth‐line therapy, and he was in good overall condition. After one session of treatment, the tumor shrank significantly in comparison to the baseline partial response (2).

#### 4.1.6 Pertuzumab

In clinical trials, the epidermal growth factor receptor (EGFR) inhibitor gefitinib (Iressa) showed anti‐cancer effectiveness against malignancies such as non‐small cell lung cancer and HNSCC. Factors that may predict response to gefitinib have been discovered through research on non‐small cell lung cancer. The molecular markers that may predict response to gefitinib in HNSCC patients are poorly understood. Erjala et al. investigated putative connections between the gefitinib responsiveness and molecular markers of the EGFR/ErbB receptor family signaling pathway. Clonogenic survival experiments were used to establish the IC50 of gefitinib sensitivity. Western and real‐time reverse transcription‐PCR analyses of EGFR, ErbB2, ErbB3, and ErbB4 expression levels, as well as phosphorylation study of pEGFR, pErbB2, pErbB3, pAkt, and pErk, were used to characterize ErbB signaling. cDNA sequencing and real‐time PCR were used to assess EGFR kinase domain sequences and EGFR gene copy numbers, respectively. Finally, the gefitinib response was compared to the anti‐EGFR antibody cetuximab (Erbitux). They found that In HNSCC, EGFR amplification may predict gefitinib susceptibility. Other members of the EGFR/ErbB receptor family may have a role in gefitinib resistance. ErbB2 and ErbB3 may be prognostic markers and therapeutic targets in treating HNSCC with gefitinib in combination therapy. According to their findings, in HNSCC, EGFR amplification may predict sensitivity and increase ErbB2/ErbB3 signaling resistance to the EGFR TKI gefitinib.

In HNSCC, the majority of targeted treatments have focused on inhibiting EGFR. Combining EGFR inhibitors with medications that target other members of the ErbB family, such as pertuzumab, may provide further benefit to HNSCC targeted therapy. The findings suggest that analyzing ErbB receptor signaling characteristics may provide predictive information and that simultaneous targeting of EGFR and other ErbB family members may reduce HNSCC cell proliferation (Table 1).

### 4.2 Anti‐human epidermal growth factor receptor 2 (anti‐HER2)

Studies have shown that the HER2 protein is highly expressed in most esophageal SCC. Human epidermal growth factor receptor 2 (HER2)/neu amplification or overexpression has also been an independent prognostic factor in Esophageal SCC (ESCC) patients. It was also noted that patients with HER2/neu amplification or overexpression might benefit from using Mabs as adjuvant therapy.^71^

#### 4.2.1 Trastuzumab

HER2 is a tyrosine‐kinase protein.^72^ HER2 plays a remarkable role in tumorigenesis. Its overexpression has been reported in many cancers, such as head and neck cancers.^73^ Moreover, HER2 promotes cancer cell development and causes tumor cells’ resistance to radiotherapy and chemotherapy that leads to poor prognosis and disease progression.^74^ Therefore, inhibiting HER2 activity through the administration of trastuzumab, a highly purified recombinant DNA‐derived IgG1 kappa Mab binding specifically to the extracellular domain of HER2 receptor, would provide a new promising approach to cancer therapy.^72^ To date, extensive studies have been designed to evaluate the efficiency of numerous combinations of trastuzumab with other treatments. Immunocytochemistry analysis demonstrated that trastuzumab restricts HER2 overexpression and stops cancer development in patients with ESCC.^75^ Supporting previous results, Yamazaki et al. showed that this combination has significant antitumor effects on all four‐cell lines of SCC.^74^

Therefore, using gefitinib and trastuzumab together would be a valuable and promising approach in HNSCC.

### 4.3 Anti‐insulin‐like growth factor type 1 receptor

The role of IGF‐1R, IGF‐1, and IGF‐2 signaling in the development, maintenance, and progression of cancers is well established.^76^ IGF‐1R expression is vital for anchorage‐independent cell growth, an essential feature of malignant cells. It can be found in most solid and hematological cancers.^76^

#### 4.3.1 Cixutumumab

Cixutumumab is a fully human Mab targeting IGF‐1R, which is involved in cancer development. Few studies on the efficiency of cixutumumab monotherapy and its combination with other methods demonstrated that this regimen shows no reliable restrictive effect on R/M HNSCC.^77^, ^78^

### 4.4 Hepatocyte growth factor/mesenchymal‐epithelial transition factor (HGF/MET) signaling inhibitors

HGF/Met signaling is associated with oncogenesis and tumor progression in different malignancies, and it induces aggressive cellular invasiveness, which is related to tumor metastasis.^79^ Therefore, the HGF receptor, tyrosine kinase MET, is a promising target in cancer treatment.^80^

#### 4.4.1 Onartuzumab

Onartuzumab is a humanized and monovalent Mab that targets MET receptors and inhibits the signaling of hepatocyte growth factor (HGF).^81^ In a phase II study, Hirsch et al. could not approve onartuzumab as a promising treatment for patients with advanced squamous cell non‐small‐cell lung cancer since the study showed no reliable anti‐tumor activities with various adverse effects such as pulmonary embolism and neutropenia.^82^ However, further studies are required to examine the efficacy of onartuzumab in a clinical setting.

#### 4.4.2 Ficlatuzumab

Ficlatuzumab is a potent humanized IgG1 Mab that binds the hepatocyte growth factor (HGF inhibitor), preventing the activation of the c‐Met pathway. It restricts HNSCC by restraining the proliferation and relocation of cancer cells and suppressing activation; therefore, based on this theory, Kumar et al. evaluated the impact of this novel Mab and concluded that ficlatuzumab viably mitigates stromal impacts on HNSCC progression.^83^ Another study showed that ficlatuzumab, in combination with cetuximab has an appropriate safety profile and demonstrates promising cytotoxic effects in a refractory population of HNSCC patients.^84^, ^85^ In conclusion, targeting C‐Met by ficlatuzumab seems a potentially promising method to manage SCC, according to recent studies.

### 4.5 Anti‐vascular endothelial growth factor (anti‐VEGF)

VEGF expression has been correlated to tumor invasion, venous and lymphatic invasion, lymph node metastasis, and tumor stage in ESCC. Therefore, it may be involved in the progression of esophageal carcinoma via its angiogenesis and lymphangiogenesis effects.^86^

#### 4.5.1 Bevacizumab

Bevacizumab, a humanized IgG1 Mab, functions as an anti‐VEGF recombinant Mab with the affinity to bind all VEGF‐A isoforms.^17^ The primary mechanism of bevacizumab activity includes binding to VEGF‐A and preventing it from joining VEGFR1 and VEGFR2.^17^, ^19^ Bevacizumab has proven beneficial therapeutic properties in the treatment of different cancers, including recurrent cervical,^87^ colorectal,^88^ renal‐cell,^89^ breast.^90^ VEGF expression correlates to tumor invasion, venous and lymphatic invasion, lymph node metastasis, and tumor stage in ESCC. Therefore, it may be involved in the progression of esophageal carcinoma via its angiogenesis and lymphangiogenesis effects.^86^ Adding a particular regimen of bevacizumab to chemotherapy and radiation results in a 95% positive response with relatively equal toxicity to other modalities, which confirmed the efficacy of adding bevacizumab and erlotinib to standard first‐line treatment.^91^

Besides, A middle‐eastern study proved bevacizumab as a promising treatment for HNSCC due to its negative effects on VEGF secretion.^92^

In addition, a combination of bevacizumab with cisplatin and radiation therapy improved the 2‐year OS and PFS with a few adverse events, such as a significant increase in myelosuppression and leukopenia.^93^

In a phase II study, adding bevacizumab to pemetrexed in patients with HNSCC enhanced median OS to 11.3 months and treatment rate to 30%; however, toxic outcomes of bevacizumab application included significant bleeding in 15%, neutropenia in 10%, and infection in 12.5% of the patients and one death due to sepsis.^94^ On the other hand, a phase I study on 10 patients with stage 4 HNSCC that received bevacizumab before chemoradiation, confirmed encouraging efficacy via tumor imaging with the most minor adverse effects.^95^ Therefore, Bevacizumab has been proven efficacious in treating HNSCC owing to a high percentage of OS with acceptable toxicity observed in different studies.^91^, ^93^, ^94^, ^95^

Twelve patients with Vulvar Squamous Cell Cancer (VSCC) were recommended targeted therapy and received one or more of bevacizumab, erlotinib, or pembrolizumab. The duration of therapy was 7 months. 10–15 mg/kg of bevacizumab was given intravenously every 3 weeks. The outcomes were CR: 22.2%, SD: 33%, and also TRAEs were seen in 75% of patients.^96^

### 4.6 Dual targeting antibodies

It has been reported that membranous HER3 expression is significantly related to poor prognosis in HNSCC patients. Thereby, it may be used as a potential target for immunotherapy.^97^

#### 4.6.1 MEHD7945A (Duligotuzumab)

Duligotuzumab is a human IgG1 dual HER3/EGFR binding Mab for overcoming drug resistance due to the extensive crosstalk between ErbB/HER receptors. It has also been reported that membranous HER3 expression is significantly related to poor prognosis in HNSCC patients. Thereby, it may be used as a potential target for immunotherapy.^97^ De Pauw et al. investigated the efficiency of Duligotuzumab with and without cisplatin in cetuximab‐sensitive and resistant HNSCC cell lines; ultimately, the results proved this regimen as a practical option in the treatment of cetuximab‐resistant HNSCC patients.^98^ On the other hand, a randomized phase II study showed that Duligotuzumab has low efficiency in the R/MHNSCC treatment outcomes.^99^ Putting an endpoint to controversies, recent studies on this dual antibody demonstrated that Duligotuzumab has positive effects on ionizing radiation.^100^

#### 4.6.2 DT‐IgG

For higher efficiency in cancer therapy, scientists aimed to design antibodies that can simultaneously affect more than one receptor.^101^ One of these antibodies is DT‐IgG, a fully humanized dual target antibody to EGFR and VEGF that restricts key growth factors.^102^ Supporting previous studies, in vivo and in vitro studies demonstrated that DT‐IgG suppresses cancer cell growth and has significant antiangiogenic effects by targeting EGFR and VEGF.^101^ Therefore, these results suggest that DT‐IgG is a helpful medication with enhanced clinical benefits in cancer therapy.

### 4.7 Anti‐CD44v6

CD44 is a transmembrane glycoprotein. Overexpression of CD44v6 shows poor prognosis in colorectal cancer, as patients with many CD44v6‐positive cells in their tumors are generally diagnosed at late stages. Anti‐CD44 therapy has good efficacies for a variety of cancers.^103^ CD44 gene encodes type 1 transmembrane glycoproteins. Specific CD44 variant isoforms, inappropriate those containing CD44 variant domain 6 (CD44v6), have been involved in tumorigenesis, tumor cell invasion, and metastasis.^104^

#### 4.7.1 Bivatuzumab

Bivatuzumab mertansine (BIWI 1) is a new CD44v6‐targeting humanized Mab with the toxin mertansine.^105^ CD44v6 antigen is expressed in HNSCC and the normal squamous epithelium.^106^ BIWI 1 has a potent antimicrotubular agent attached to a Mab against CD44v6.^106^ Reichmann et al.'s study on adult patients with R/MHNSCC, treated with BIWI 1 intravenously, confirmed that by CD44v6 expressing tumors, bivatuzumab has direct mertansine activity (Table 3).^106^

## 5 CHECKPOINT INHIBITORS

Immune checkpoints that are in interaction with their ligands, inhibit T cell activation pathways that are responsible for physiological immune responses to certain antigens. Various malignancies have high levels of immune checkpoints, and their ligand in the tumor microenvironment, which leads to blockage of the induction of appropriate anti‐cancer immune responses. Several immune checkpoint inhibitors have been developed, examined, and approved for treating various malignancies.^107^

### 5.1 Anti‐PD‐1

PD‐1 is predominantly expressed in T cells. Its interaction with PD‐L1 and PD‐L2 indicated in antigen‐presenting cells (APCs) and tumors sends a negative signal to T cells, which can cause T‐cell exhaustion.^108^

#### 5.1.1 Nivolumab

PD‐1, a cytotoxic T‐cell response inhibitor molecule, contributes to skin cancer development.^109^ It is often highly expressed in CSCC.^110^, ^111^ Antibodies targeting CTLA‐4 and PD‐1 display clinical effectiveness in metastatic melanoma and Merkel cell carcinoma.^112^, ^113^, ^114^ Nivolumab, a fully human IgG4 Mab to inhibit PD‐1, hinders the pathway, which prohibits activated T cells from cancer‐fighting, consequently helping the immune system eliminate the tumor.^115^ Since HNSCC recurrence and metastasis are allowed by immune avoidance, T‐cell suppression by PD‐1 blockage represents potential clinical efficacy against these tumors.^116^ Expression of PD‐L1 is enhanced in ESCC, which may sensitize the tumor to eradication by immune checkpoint inhibitors.^117^

Nivolumab is a human IgG4 Mab that blocks the interaction between the PD‐1 receptor and PD‐L1 and PD‐L2 via binding to the PD‐1 receptor, leading to inhibition of T cell cytokine production and proliferation. A phase III study compared the effect of nivolumab and chemotherapy on unresectable recurrent or advanced ESCC. It demonstrated that nivolumab significantly improved the OS and had an acceptable safety profile.^117^ As an outcome of this result, the positive effects of nivolumab on OS in patients with R/MHNSCC have been proved earlier.^118^ In addition, a study on a patient with CSCC showed that complete remission was achieved within 6 months of immunotherapy with cetuximab and nivolumab.^119^ In the case of cost‐effectiveness, studies have argued that although nivolumab increases OS, it is not a cost‐effective option for treating R/MHNSCC.^120^, ^121^

In a study conducted by Yamakawa et al., 143 patients were treated with nivolumab. 27.3% of patients showed overall response and the disease was controlled in 46.2% of them. The overall survival of the patients was 11.2 months and the progression‐free survival rate was 2.7 months.^122^

Matsubara et al. conducted a study to predict the efficacy of Nivolumab on esophageal SCC by combined positive score (CPS) and tumor proportion score (TPS). The mean progression‐free survival (3.2, 2.5, and 1.5 months) and objective response rate (30%, 25%, and 0%) of patients with CPS ≥10, 1–10, and <1 had a downward trend, respectively.^123^

#### 5.1.2 Pembrolizumab

In multiple tumor forms, pembrolizumab, a highly selective humanized IgG4 Mab to PD‐1 receptor, has demonstrated robust antitumor effects and a tolerable safety profile and is currently validated for one or even more progressive tumors treatment in more than 60 countries worldwide.^124^ Several studies show that pembrolizumab provides better outcomes for patients with CSCC by targeting PD‐1.^125^ Moreover, a recent study supported this theory and showed the important survival advantages of pembrolizumab compared to usual treatments.^126^ Shah et al. proved that pembrolizumab could be a promising and safe treatment for patients with metastatic ESCC.^127^ Further studies on the monotherapy of pembrolizumab (or combined with cetuximab and chemotherapy) showed that this Mab has an acceptable efficiency and safety profile.^128^ Therefore, pembrolizumab would make the first‐line therapy for PD‐L1‐positive chronic or metastatic HNSCC with the least concerns in the future.

Phase Ib KEYNOTE‐012 research showed that treating R/MHNSCC cases with particular pembrolizumab regimens (10 mg/kg every 2 weeks or 200 mg every 3 weeks) shows the highest survival rate and safety.^124^ Also, the fixed dosage of pembrolizumab has been well accepted, yielding a better clinical objective response rate (ORR) with evidence of reliable reactions, which further supports this treatment.^129^ Therefore, using pembrolizumab in patients with PD‐L1‐positive chronic or metastatic HNSCC can provide better outcomes in the future.^130^ The last reports of this cohort demonstrated that 200 mg every 3 weeks of pembrolizumab had the best acceptance between cases and resulted in successful outcomes.^131^ In conclusion, various studies have to date, lightened many aspects of pembrolizumab efficiency, safety, and regimen and introduced this treatment as a promising option for treating patients with SCC.

Ferrarotto et al. evaluated the effects of pembrolizumab in 19 patients with advanced, refractory CSCC. The patients had a long‐term follow‐up of 44.1 months. The results indicated pembrolizumab to be effective and safe in this population; However, two patients (10%) experienced remarkable treatment‐associated side effects.^132^

One research paper evaluated 628 Asian patients with advanced or metastatic ESCC to compare the therapeutic effects of pembrolizumab with chemotherapy. There were 314 patients in each treatment group. The Pembrolizumab group demonstrated survival advantages over patients who were treated with chemotherapy. Additionally, Fewer treatment‐related complications emerged in the pembrolizumab‐treated patients, which confirmed the safety superiority of pembrolizumab over chemotherapy.^133^

In another study, a regimen including pembrolizumab and cetuximab was administered to 33 patients with metastatic or recurrent HNSCC. This combination resulted in promising clinical advantages with a 6‐month ORR of 45%, which exceeded the previously published ORR of monotherapy with each antibody. Of note, 15% of the patients experienced severe adverse effects. The most common grade 3 or higher side effect was oral mucositis in 9% of the patients. Five patients stopped the treatment due to side effects.^51^

One phase II study included 159 patients with locally advanced or metastatic/recurrent CSCC from 59 centers. Every 3 weeks, the patients have treated with pembrolizumab 200 mg intravenously, repeated for the utmost 35 cycles. The immediate, potent anti‐tumor effects of pembrolizumab and its manageable toxicity, portrayed pembrolizumab as a promising treatment option in these patients.^134^

In an attempt to profile the biomarker aspects of pembrolizumab therapy, Haddad et al. investigated the correlations between certain biomarkers and clinical outcomes of pembrolizumab therapy in 192 patients with metastatic/recurrent HNSCC. Among the quantified biomarkers, tumor mutational burden (TMB), PD‐L1, and 18‐gene T‐cell‐inflamed gene expression seemed to have the potential as predictive biomarkers of patients’ response to pembrolizumab.^135^

However, combining the treatment of pembrolizumab with Acalabrutinib failed to increase the efficacy of pembrolizumab monotherapy. The related toxicity of this combination was another obstacle that seemed to hinder the feasibility of this treatment.^136^

Cohen et al. applied pembrolizumab with intralesional SD‐101 in a population of recurrent/metastatic HNSCC patients. The treatment combination prompted the objective response, especially in patients who were human papillomavirus positive. The results could be due to enhanced inflammation and activity of effector immune cells inside the tumor.^137^

In a recent phase II study, the combined therapy of pembrolizumab with afatinib was tested. Additionally, a biomarker quantification was also performed. Twenty‐nine patients with recurrent or metastatic HNSCC whose disease was refractory post platinum therapy were included. The conclusion suggested that afatinib may enhance pembrolizumab efficacy with improved ORR. Also, antigen presentation and cytotoxicity induced by natural killer cells (NK cells), indicated upregulation in the tumor tissue. Nonetheless, treatment toxicity of grade ≥3 was reported in 37.9% of the cases, which can be an issue of concern in applying this combination.^138^

A study performed by Tao et al., to compare the effect of pembrolizumab and cetuximab in the treatment of locally advanced SCC of the head and neck. Seventy five percent of patients had metastases. There was no significant difference between the locoregional control rate (*p* = .91), progression‐free survival (*p* = .47), and overall survival (*p* = .49) of the two groups. But the toxicity in pembrolizumab treatment was lower than cetuximab.^139^

#### 5.1.3 Durvalumab

Durvalumab is a human IgG1 kappa Mab blocking PD‐L1 on malignant cells from binding to PD‐1 and CD80 on T cells, subsequently allowing higher T cell activation and anti‐cancer activity.^140^, ^141^ The first studies on anti‐PD‐L1 agents showed that Durvalumab significantly improves OS and PFS in R/MHNSCC cases with a few acceptable adverse effects.^142^ Despite appropriate outcomes of applying durvalumab for HNSCC cases in the phase I/II study, Segal et al. reported several adverse events, such as fatigue, diarrhea, and nausea; therefore, much attention has been attracted to controlling the adverse effects of this treatment.^143^ On the other hand, parallel studies have shown that combining Durvalumab with tremelimumab could ameliorate outcomes even in cases with <25% PD‐L1 expression.^144^ In another study, Ferris et al. demonstrated that this combination has a higher response rate and survival, with no significant difference in OS.^18^

Taken together, due to the high survival of patients using durvalumab, it is a novel promising treatment for HNSCC not only in patients with high PD‐L1 profiles but also in patients with low PD‐L1 manifestations.^142^, ^144^

In patients with esophageal squamous cell carcinoma, Park et al. looked at the efficacy of adjuvant durvalumab after neoadjuvant concurrent chemoradiotherapy (CCRT) (ESCC). In patients with ESCC, they were unable to show that adjuvant durvalumab enhanced survival following neoadjuvant CCRT. However, post‐CCRT PD‐L1 expression may predict survival in patients who get adjuvant durvalumab following neoadjuvant CCRT, although it needs to be shown. The use of adjuvant durvalumab was generally safe in this research, with events equivalent to those seen in prior durvalumab monotherapy studies. Only three individuals had to stop taking the study medicine permanently due to side effects, most of which were minor. In conclusion, the current placebo‐controlled, double‐blind, randomized phase II study failed to fulfill the primary goal, likely due to the small sample size and the control arm's outperformance. They did, however, gain important information for future study design. First, after neoadjuvant CCRT and surgery, adjuvant durvalumab therapy appears to be safe. Second, patients who receive adjuvant PD‐1/PD‐L1 inhibitors may benefit from knowing their post‐CCRT PD‐L1 status. Third, because patients with ypCR have a favorable prognosis and are less likely to benefit from adjuvant durvalumab, future studies studying adjuvant immunotherapy should be cautious when enrolling this population. They are cautiously optimistic that the efficacy of adjuvant ICIs, such as durvalumab, in patients with esophageal cancer will be confirmed in larger investigations.

On the other hand, multiple clinical trials evaluate durvalumab, a programmed cell death ligand‐1 (PD‐L1)‐blocking monoclonal antibody, for treating HNSCC. Arends et al. looked at circulating proteins at the start of the study to see if there were any relevant biomarkers and to figure out what pathways were linked to durvalumab's clinical outcomes. For 158 durvalumab‐treated HNSCC patients in the phase II HAWK and CONDOR trials as a discovery dataset and 209 durvalumab‐treated HNSCC patients in the phase III EAGLE study as a validation dataset, 66 serum proteins were evaluated using multiplex immunoassays before therapy. Higher baseline concentrations of interleukin‐6 (IL‐6), C‐reactive protein, S100 calcium‐binding protein A12, and angiopoietin‐2 (ANGPT2) were associated with shorter overall survival (OS) after durvalumab treatment (p.05), in comparison higher concentrations of osteocalcin were associated with longer OS after durvalumab treatment (p.05). After correcting for baseline clinical variables, all five proteins remained strongly linked with OS, with consistent results across clinical efficacy endpoints based on univariate correlation analysis. The EAGLE trial's validation dataset validated the independent associations of IL‐6 and osteocalcin with OS and preserved directional patterns for the other biomarkers discovered in the discovery dataset. Their findings show that immunosuppressive proteins play a key role in HNSCC resistance to durvalumab treatment. There was a positive link between osteocalcin and clinical outcomes, which needs further examination. In conclusion, in durvalumab‐treated patients with HNSCC, circulating IL‐6 and osteocalcin concentrations at baseline have been demonstrated to be independently linked with clinical outcomes, with trends of additional effects for ANGPT2 in three clinical studies. In clinical trials of ICI monotherapy and combination therapy, measurement of these biomarkers before and after treatment should be the priority.^145^, ^146^

#### 5.1.4 Avelumab

Avelumab is a fully human IgG1 lambda Mab against PD‐L1 that has shown a ADCC activity.^147^ In a study by Elbers et al., a combination of avelumab with cetuximab‐RT showed in patients with advanced‐stage HNSCC. In this study, it has been shown that avelumab plus cetuximab‐RT is a possible therapy for patients who are not suitable for cisplatin treatment and also have advanced‐stage HNSCC.^148^

Guigay et al. examined the effect of avelumab, an anti‐programmed death ligand 1 antibody, in metastatic or refractory head and neck squamous cell carcinoma (R/M SCCHN). Patients were aged ≥18 years and had previously received platinum‐based chemotherapy. Within 6 months of treatment, some were ineligible for platinum‐based chemotherapy, and some had refractory SCCHN. A dosage of 10 mg/kg every 2 weeks of avelumab was given to patients. The efficacy and safety of avelumab were reported and had a low rate of grade ≥3 TRAEs in R/M SCCHN and heavily pretreated patients.^149^

#### 5.1.5 Cemiplimab

Cemiplimab, a high‐affinity IgG4 Mab, blocks the PD‐1/PD‐L1 pathway.^150^ The treatment of CSCC with cemiplimab is more efficacious compared to platinum‐based chemotherapy in terms of OS.^151^ Gross et al. proved that treating stage III/IV (M0) CSCC‐HN cases with two doses of cemiplimab 350 mg, every 3 weeks before surgery shows no grade 3 or 4 adverse events. They also reported 55% pathologic complete response and 15% other major pathologic response (NPR, ≤10% viable tumor). Therefore, these results suggest cemiplimab as a potential neoadjuvant therapy.^152^

Migden et al. reported a response of 50% in a phase 1 study of cemiplimab for expansion cohorts of patients with locally advanced or metastatic CSCC with 26 patients and 47% in a pivotal phase 2 study of cemiplimab for a cohort of patients with metastatic disease with 59 patients.^153^

In a study made by Rischin et al.,^154^ patients with advanced cutaneous squamous cell carcinoma: metastatic (mCSCC) and locally advanced (CSCC) were evaluated. Patients were derived into three groups, group 1 (mCSCC) and 2 (CSCC) received 3 mg/kg of cemiplimab every 2 weeks, and group 3 received 350 mg every 3 weeks. ORR per ICR was 46.1%, and the median duration of follow‐up was 15.7 months. CR rates were 20.3%, 12.8%, and 16.1% for groups 1, 2, and 3. OS estimated by Kaplan–Meier was 73.3%, but the median OS was unavailable. Finally, Grade ≥3 TRAEs were seen in 17.1% of patients.

In a phase I trial, Babiker et al. studied 15 patients who received combined therapy of cemiplimab, cyclophosphamide, radiation therapy (RT), and granulocyte‐macrophage colony‐stimulating factor1 (GM‐CSF). Mostly seen TEAEs were fatigue, constipation, asthenia, dyspnea, maculopapular rash, and pneumonia. Only two patients faced grade ≥3 TEAE (pneumonia). The efficacy of this combination therapy was not above other PD‐1 inhibitor monotherapies in R/M SCCHN.^155^

Treatment with Cemiplimab‐rwlc (CEMI) in metastatic or locally advanced CSCC has demonstrated its efficacy. Eighteen patients were evaluated in a study, and 3 mg/kg every 2 weeks or 350 mg every 3 weeks infusions of CEMI were given to the patients. Median follow‐up was 8 months, ORR was 67%, CR was 33%, PR was 33%, and DCR was 72%. Thirty three percent of adverse events with grades 2, 3, and 5 were observed in six patients.^156^

A study was conducted by Bailly‐Caille et al. on advanced cutaneous SCC, in which the therapeutic effect of Cemiplimab alone and in combination with radiotherapy was investigated. In this study, the observed objective response rate was 47.6 in Cemiplimab consumption versus 41.6 in combined treatment. The simultaneous use of radiotherapy and Cemiplimab (3 months) caused a faster clinical‐radiological response than the use of Cemiplimab alone (5.5 months).^157^

In a study by Ríos‐Viñuela et al., 13 patients with advanced cutaneous SCC were treated with Cemiplimab. 23% of patients showed complete response and 38% partial response to treatment. 80% of patients with partial response experienced disease progression during follow‐up. 46% of patients showed mild side effects.^158^

A phase 2 study was conducted by Gross et al. to investigate the effect of neoadjuvant therapy of cemiplimab in cutaneous SCC. 51% of patients showed a complete pathological response and 13% of them showed a major pathological response, that is, less than 10% of tumor cells remaining. Also, objective response was reported in 68% of patients.^159^

### 5.2 Anti‐CTLA

CTLA‐4, expressed on T cells, interacts with CD80/CD86, limiting T‐cell activation and leading to anergy.^108^ CTLA‐4 is a CD28 homolog with a high affinity for B7‐1/2. While the CD28:B7‐1/2 interaction serves as a co‐stimulatory signal for T cell proliferation and activation, the CTLA‐4:B7‐1/2 binding acts as a co‐inhibitory signal to thwart early T cell activation.^160^

#### 5.2.1 Ipilimumab

The combination of chemotherapy as the first line therapy with ipilimumab, a human IgG1 kappa antibody to CTLA4, has not resulted in significant changes in survival^161^; therefore, attention was attracted to using this treatment combined with other agents as a second line therapy. For the first time in a study, a combination of ipilimumab with programmed death (PD)‐1 inhibitor nivolumab, a known melanoma treatment approved by the FDA, had successful results in treating a patient with refractory HNSCC after 4 months; on the other hand, examining the level of PD ligand‐1 is required until we can use this method as a proved treatment.^162^ A novel study, designed in 2020, showed the successful effects of this combination before the surgery in patients with oral cavity squamous cell carcinoma (OSCC).^163^

Altogether, more studies are required to find new methods and to consider the side effects of ipilimumab, to prove safer approaches.

#### 5.2.2 Tremelimumab

Tremelimumab is a fully human IgG2 Mab that targets cytotoxic T‐lymphocyte‐associated antigen‐4 (CTLA‐4) and exhibits antitumoral effects by blocking the CTLA‐4 pathway.^164^

Phase II research investigated the efficiency of tremelimumab as monotherapy. There were about 244 patients in each group. One group received durvalumab as monotherapy (group 1), the second group received both tremelimumab and durvalumab as combined therapy, and the third group was standard of care (SoC). The results showed no marked differences in OS between durvalumab monotherapy or combined use of durvalumab and tremelimumab versus SoC. However, survival rates increased in 12–24 months, and response rates showed that durvalumab was a beneficial therapeutic agent.^18^ Ferris et al. conducted a study to investigate the effect of using Durvalumab with or without tremelimumab in patients with recurrent or metastatic head and neck SCC. The results showed that OS did not significantly differ in the case of durvalumab monotherapy or durvalumab plus tremelimumab combination therapy compared to SoC.^18^ A phase 2 study was also conducted on durvalumab (D), tremelimumab (T) monotherapy, and combined durvalumab and tremelimumab therapy (T + D). The study showed acceptable toxicity in pretreated, PD‐L1 low/negative, and R/M HNSCC patients, and no side effects were observed.^165^

## 6 ANTI‐NK INHIBITORY RECEPTOR CD94/NK GROUP 2 MEMBER (ANTI‐NKG2A)

A key mechanism of tumor resistance to immune cells is mediated by the expression of peptide‐loaded HLA class I molecule (HLA‐E) in tumor cells, which suppresses NK cell activity via ligation of the NKG2A.^166^ NKG2A blockade, in combination with other therapeutic antibodies showed encouraging responses in a subset of patients with metastatic colorectal or head and neck cancer. However, established biomarkers of response are lacking, and larger trials are needed to enable firm conclusions about whether NKG2A inhibition complements existing immunotherapies.^167^

### 6.1 Monalizumab

Monalizumab is the first‐in‐class humanized IgG4 Mab targeting NKG2A, which enhances NK cell activity against various tumor cells and rescues CD8+ T cell function. It also stimulates NK cell activity against antibody‐coated target cells.^168^ The combination of monalizumab and cetuximab reduced almost all cetuximab‐mediated side effects and showed more reliable outcomes.^169^

## 7 TUMOR TARGETING: MAB U36 AND MAB E48

**FIGURE 3:**
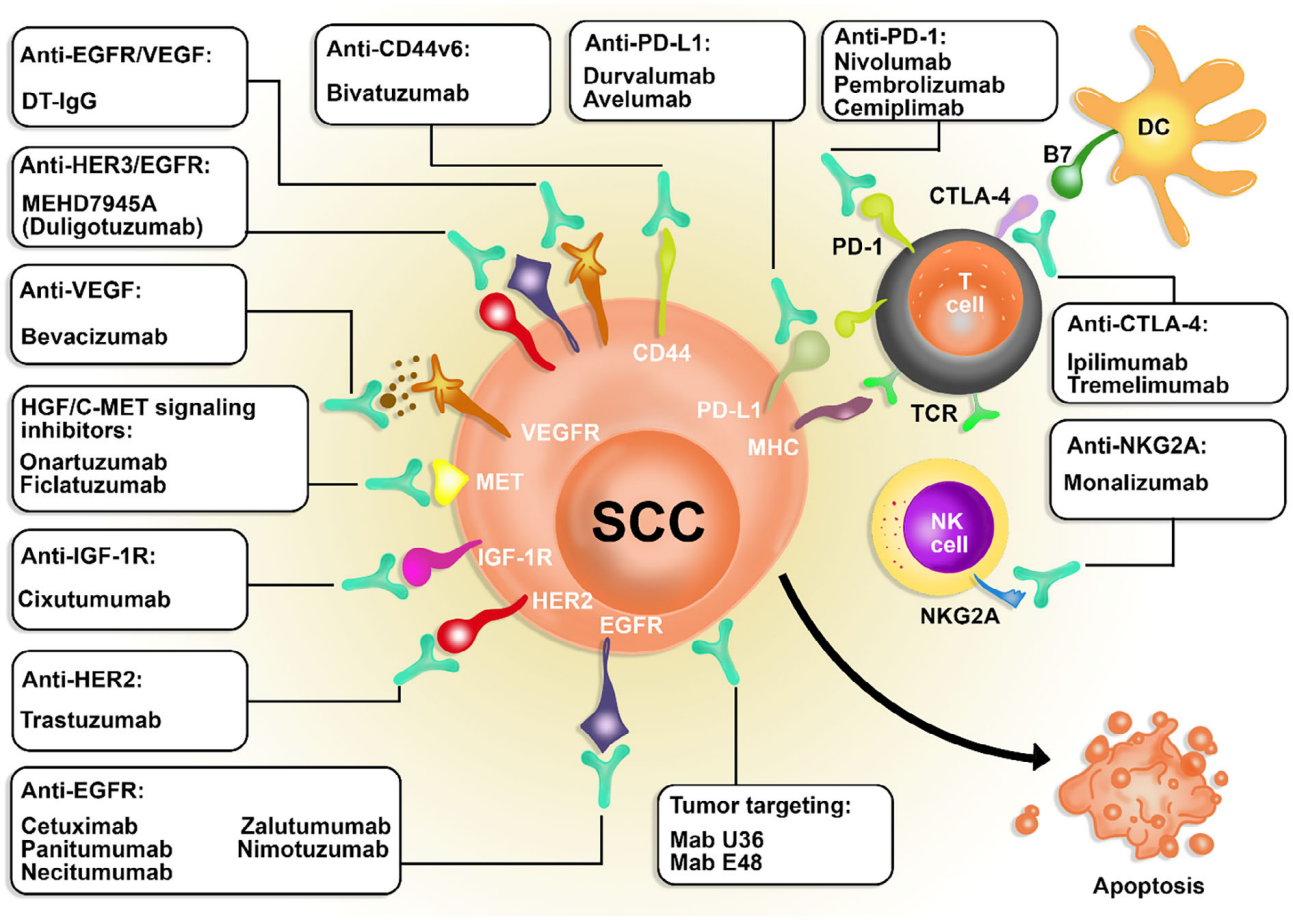
Monoclonal antibodies (Mab) for treatment of Squamous cell carcinoma (SCC). Antibodies to receptors/ligands, checkpoint inhibitors, and tumor targeting Mab U36 and E48 are the three main types of Mabs in SCC therapy.

Using cMab U36 labeled with 211Astatine showed reliable results in treating rats with HNSCC; on the other hand, combining this radioimmunotherapy with cetuximab revealed better results in controlling carcinoma and drew attention to interactions between CD44v6 and epidermal growth factor receptor (EGFR).^170^ Other possible underlying mechanisms of action are yet to be discovered (Figure 3 summarizes all the monoclonal antibodies used for treating SCC).

## 8 DISCUSSION

Various Mabs, working through various mechanisms, have been studied in treating the SCC of different organs, and different results have been found. The main groups of Mabs in SCC therapy are Mabs targeting EGFR, HER2, HGF, and VEGF, alongside checkpoint inhibitors (including Mabs to PD‐1, CTLA4, and NKG2A) and tumor targeting Mabs. Here, we discuss some of the most crucial roles of Mabs in SCC therapy according to what was mentioned earlier in the study, by Mab, and by organ:

### 8.1 Anti‐EGFR monoclonal antibodies

Most HNSCCs express high levels of EGFR.^43^, ^44^ Among Anti‐EGFR Mabs, cetuximab (a highly studied Mab in SCC treatment) such as zalutumumab and panitumumab has shown favorable outcomes in locoregionally advanced, recurrent, or metastatic HNSCC, specifically HPV‐negative SCCs.^49^, ^50^, ^53^, ^55^, ^56^, ^57^, ^58^, ^60^, ^171^, ^172^, ^173^ Cetuximab is also helpful in sensitizing the tumor to radiotherapy.^45^, ^46^, ^47^ Cetuximab showed some grade 3 and 4 adverse effects such as folliculitis, anemia, trichomegaly,^48^ acneiform rash and infusion,^45^ neutropenia,^50^ and hyper transaminasemia^174^ with different doses of chemo‐ and radiotherapy. However, recent studies have shown that cetuximab cannot develop any significant adverse effects. It also can improve the safety of other antitumor therapies. Ham et al. revealed that combining methotrexate with cetuximab would significantly decrease the adverse effects.^49^ Also, further trials showed that using higher doses in the first days and continuing with lower doses would ameliorate the chance of different local adverse effects.^175^

Another anti‐EGFR Mab, nimotuzumab has been proven to induce promising results in SCC of different organs, including the head and neck, lung, and esophagus. It may be considered a first‐line treatment in these patients.^61^, ^62^, ^63^, ^64^, ^65^, ^66^ High doses of nimotuzumab would provide broad adverse effects on the skin, gastrointestinal tract, and general symptoms,^62^ like chills and fever.^61^ Recent studies reported that administering lower doses in the first days and gradually increasing the doses (in 2 or 3 weeks) with or without chemo‐ and radiotherapy ameliorated the adverse effects of nimotuzumab.^63^, ^176^

### 8.2 Monoclonal antibodies to overcome anti‐EGFR resistance

Trastuzumab, an anti‐HER2 Mab, can induce ADCC in ESCCs with high HER2 expression, especially in combination with cetuximab.^74^, ^75^ Cixutumumab, onartuzumab, duligotuzumab, and DT‐IgG have not shown much significant benefit in SCC treatment; further investigation is needed to evaluate their potential efficacy, especially in overcoming cetuximab resistance, in different combinations and special clinical settings.^77^, ^78^, ^82^, ^99^, ^100^, ^101^ Trastuzumab is one of the safest Mabs in this group. This treatment showed dramatic tolerability in different regimens, such as monotherapy,^75^ and in combination with cetuximab^74^ or gefitinib.^177^ As a result, trastuzumab would be one of the most promising Mab therapies with the least adverse effects and high efficiency.

Ficlatuzumab, an anti‐HGF Mab, can promote cytotoxicity in combination with cetuximab in refractory HNSCC. Nonetheless, it surely needs more investigation to evaluate its effectiveness and toxicity in clinical trials.^84^, ^85^, ^178^

### 8.3 Checkpoint inhibitors

Positive outcomes in treating SCCs with PD‐1 inhibitors have been illustrated, probably due to the high expression of PD‐L1 in ESCC, CSCC, and HNSCCs.^110^, ^111^, ^116^, ^117^ Among them, nivolumab, through the improvement of survival and comparable safety, shows efficacy treatment of ESCC.^117^ It has also yielded acceptable survival improvement in HNSCC cases; however, it may not be a cost‐effective choice in this setting.^120^, ^121^, ^179^ Another potent Mab in the treatment of SCC is pembrolizumab, which has promoted cytotoxicity in combination with chemotherapy, and it has a good safety profile. Thus, it appears to be a suitable first‐line therapy in R/M HNSCC, especially for heavily pretreated patients.^128^, ^130^, ^131^ It may also be used in locally progressed CSCC.^125^

Avelumab, combined with cetuximab, has been observed to be an efficient treatment for advanced‐stage HNSCC patients who are unsuitable for treatment with cisplatin.^148^ Cemiplimab has induced a longer survival than chemotherapy in CSCC; it can be applied in CSCC of the head and neck as neoadjuvant therapy to achieve a less extensive surgical removal.^151^, ^152^ Durvalumab, another potent anti‐PDL1 Mab in treating R/MHNSCC, especially in HPV‐positive tumors, is efficient in both high and low PD‐L1‐expressing patients and associated with a suitable safety profile.^142^, ^143^, ^165^

Ipilimumab and tremelimumab, CTLA4 inhibitors, have shown controversial results in treating SCC. Although ipilimumab, in combination with nivolumab, has shown potential efficacy both in lung SCC and as second‐line therapy in HPV‐related HNSCC,^161^, ^162^, ^164^, ^165^, ^180^ more studies are required on their utility in the treatment of SCC. Also, further studies are required to lower SCC's common adverse effects, such as peripheral sensory neuropathy, colitis, and elevation of hepatic enzymes.^161^, ^162^ Monalizumab is an NKG2A inhibitor, similar to several other antibodies in the treatment of R/MHNSCC, and makes an efficient combination with cetuximab.^169^

### 8.4 Other monoclonal antibodies in squamous cell carcinoma therapy

Bevacizumab has higher efficiency as well as similar toxicities when compared to chemotherapy and radiotherapy. It is also a good option in combination with other modalities in advanced HNSCC therapy, even as first‐line treatment.^93^, ^94^, ^95^ Tumor targeting Mabs, Mab U36, and Mab E48, especially in combination with cetuximab, along with bivatuzumab‐mertansine combination, may be effective in HNSC^106^, ^170^, ^181^, ^182^, ^183^, ^184^ and well‐tolerated,^87^ but with a bit consideration of bleeding in some cases.^94^

### 8.5 Head and neck squamous cell carcinoma

Mabs have been widely used and studied in the treatment of HNSCC. Due to the fruitful results of using Mabs in clinical settings, they are considered to have a key role in this era, especially in the advanced stages of the disease. Mabs are considered excellent options in locally advanced, recurrent, and metastatic HNSCCs. The most potent Mabs in HNSCC are EGFR antagonists and checkpoint inhibitors, especially PD‐L1 antagonists.^49^, ^50^, ^53^, ^55^, ^56^, ^57^, ^58^, ^60^, ^116^, ^117^, ^128^, ^130^, ^131^, ^142^, ^143^, ^148^, ^165^, ^171^, ^172^, ^173^ Mabs have also been approved by FDA for treating HNSCC, including cetuximab and pembrolizumab. Cetuximab was approved as monotherapy or in combination with chemotherapy in locally or regionally advanced and recurrent or metastatic HNSCC after treatment with platinum‐based therapy.^185^ FDA has also approved pembrolizumab as the first‐line therapy of unresectable HNSCC, with a high safety observed in multiple studies.^186^

### 8.6 Esophageal squamous cell carcinoma

Outstanding results of treating ESCC with Mabs have encouraged their wide use for this cancer. PD‐1 inhibitors, especially nivolumab, have exhibited favorable efficacy in ESCC.^117^ Therefore, FDA approved nivolumab for patients with unresectable advanced, recurrent, or metastatic ESCC after fluoropyrimidine‐ and platinum‐based chemotherapy.^179^ Despite promising results in recent studies, nivolumab showed few pulmonary and gastric adverse effects in some cases.^117^, ^178^ however, the current trials improved these adverse effects dramatically.^118^ Mabs may have an important role in treating esophageal cancers in the near future, to be used either after tumor resection or in advanced tumor settings. Investigation of their cost‐effectiveness and determining response predictors are of great importance in this subject.

### 8.7 Cutaneous squamous cell carcinoma

CSCC has also shown promising responses to Mabs, especially checkpoint inhibitors.^125^ Pembrolizumab has been approved by FDA for recurrent or metastatic CSCC that is not treatableby either surgery or radiation.^187^ Nivolumab and Cemiplimab have also shown encouraging results in the treatment of CSCC.^151^, ^152^ Cemiplimab has been demonstrated to be a suitable choice as neoadjuvant therapy in stage III/IV and a solo therapy in metastatic CSCC.^152^ Using Mabs in CSCC is a potential topic in future research.

### 8.8 Lung squamous cell carcinoma

Several Mabs, including nimotuzumab, bevacizumab, ipilimumab, necitumumab, and pembrolizumab have shown acceptable efficacy in treating Lung SCC as a type of non‐small cell lung cancer.^65^, ^68^, ^161^, ^188^, ^189^, ^190^, ^191^, ^192^ FDA has approved the combination of nivolumab and ipilimumab as the first‐line treatment of metastatic non‐small cell lung cancers that express PD‐L1 with no genomic tumor aberrations in EGFR or anaplastic lymphoma kinase (ALK).^193^ Pembrolizumab has also been approved by FDA in combination with carboplatin and either paclitaxel or nab‐paclitaxel as the first‐line treatment of metastatic squamous non‐small cell lung cancer.^194^ Mabs are the potential widely used future treatment of metastatic lung SCC.

## 9 CONCLUSION AND FUTURE PERSPECTIVES

Altogether, treatments with different Mabs have shown excellent efficacy along with acceptable safety in the SCC of different organs. Therefore, Mabs are considered excellent options in treating SCC, especially for the advanced stages of the disease. Overall, two highly potent types of Mabs in SCC therapy are anti‐EGFR Mabs and checkpoint inhibitors, especially cetuximab, nimotuzumab, and PD‐1 inhibitors. Cetuximab can also be used not just as first‐line treatment in R/MHNSCC, but additionally as a second and third treatment due to its strong response rate. Bevacizumab is another beneficial option to be added to other modalities; it can indirectly decrease cell proliferation and enhance apoptosis By reducing the nutrient supply to the tumor. Furthermore, when taken with standard anticancer medicines, bevacizumab is likely to have additional antitumor effects. It was also discovered that patients with recurrent squamous‐cell carcinoma of the head and neck who received zalutumumab had longer progression‐free survival. Nivolumab has also emerged as a remarkable therapeutic option with an acceptable toxicity profile in patients with R/M HNSCC who have had postplatinum therapy. Although some Mabs have shown promising outcomes in SCC therapy, their application as a part of cancer treatment depends on further investigations on their cost‐effectiveness and predictors of response. Additionally, phase 4 trials and studies, comparing different Mabs to identify their advantages and disadvantages and choosing the best choice in each clinical setting are required. Due to significant efficacy, several Mabs are considered valuable choices in different lines of the treatment of SCC of different organs. However, some concerns still exist. Some Mabs still need more investigations in terms of safety, underlying mechanisms of action, and the extent to which they enhance treatment response. Cetuximab is a widely studied Mab that had shown severe adverse effects in first trials. However, recent studies in the present decade improved its safety by setting different combined therapies and more exact regimens.

On the other hand, some Mabs, such as bevacizumab, trastuzumab, and pembrolizumab, revealed very slight adverse effects in mono‐ and multi‐therapies. Combining these safe Mabs with more efficient but less safe ones would develop more promising regimens to adjust the adverse effects of Mab‐therapies and achieve the best antitumor treatments in the future. The high price is another obstacle to the wide use of Mabs, particularly in developing countries. The development of safe new effective methods of Mab production, Mabs to new biomarkers, and dual‐targeting Mabs may propose Mabs as a promising treatment of SCC of different organs in the future.

## AUTHOR CONTRIBUTIONS

**Amirhossein Tamimi:** Investigation (lead); methodology (equal); resources (equal); visualization (equal); writing – original draft (lead); writing – review and editing (supporting). **Atena Tamimi:** Investigation (supporting); methodology (equal); resources (equal); visualization (equal); writing – original draft (supporting); writing – review and editing (supporting). **Fatemeh Sorkheh:** Conceptualization (supporting); investigation (supporting); methodology (equal); resources (equal); visualization (equal); writing – original draft (supporting); writing – review and editing (supporting). **Saba Mardekatani Asl:** Conceptualization (supporting); investigation (equal); resources (equal); visualization (equal); writing – original draft (supporting); writing – review and editing (supporting). **Arezoo Ghafari:** Conceptualization (equal); investigation (supporting); visualization (equal); writing – original draft (supporting); writing – review and editing (supporting). **Arian Ghannadi Karimi:** Conceptualization (supporting); investigation (supporting); resources (supporting); visualization (equal); writing – original draft (supporting); writing – review and editing (supporting). **Gisou Erabi:** Conceptualization (supporting); investigation (equal); visualization (equal); writing – original draft (supporting); writing – review and editing (supporting). **hossein pourmontaseri:** Conceptualization (equal); project administration (supporting); supervision (supporting); visualization (equal); writing – original draft (equal); writing – review and editing (supporting). **Niloofar Deravi:** Conceptualization (lead); investigation (supporting); methodology (equal); project administration (lead); supervision (lead); writing – original draft (supporting); writing – review and editing (lead).

## CONFLICT OF INTEREST STATEMENT

The authors declare no conflict of interest.

## Data Availability

Data are available upon request from corresponding author.
